# Comparative Study of Focused Ultrasound Unilateral Thalamotomy and Subthalamotomy for Medication‐Refractory Parkinson's Disease Tremor

**DOI:** 10.1002/mds.30159

**Published:** 2025-03-03

**Authors:** Steffen Paschen, Elena Natera‐Villalba, José A. Pineda‐Pardo, Marta del Álamo, Rafael Rodríguez‐Rojas, Johannes Hensler, Günther Deuschl, Jose A. Obeso, Ann‐Kristin Helmers, Raúl Martínez‐Fernández

**Affiliations:** ^1^ Department of Neurology University Hospital Schleswig‐Holstein, Christian‐Albrechts‐University Kiel Germany; ^2^ Centro Integral de Neurociencias AC (CINAC) HM Universitario Puerta del Sur Madrid Spain; ^3^ PhD Medicine Program Universidad Autonoma de Madrid Madrid Spain; ^4^ Instituto de Investigación Sanitaria HM Hospitales Madrid Spain; ^5^ Department of Radiology and Neuroradiology University Hospital Schleswig‐Holstein, Christian‐Albrechts‐University Kiel Germany; ^6^ Universidad CEU‐San Pablo Madrid Spain; ^7^ Department of Neurosurgery University Hospital Schleswig‐Holstein, Christian‐Albrechts‐University Kiel Germany

**Keywords:** focused ultrasound, Parkinson' disease, tremor, subthalamic nucleus, ventrointermedius nucleus

## Abstract

**Background:**

Unilateral focused ultrasound ventral intermediate thalamotomy (Vim‐FUS) is effective in treating Parkinson's disease (PD) tremor. Ultrasound ablation of the subthalamic nucleus (STN‐FUS) has demonstrated efficacy in improving all cardinal motor features of PD, including tremor.

**Objective:**

To compare the efficacy in parkinsonian tremor control between Vim‐FUS and STN‐FUS.

**Methods:**

Retrospective, two‐center study including consecutive PD patients with medication‐refractory tremor who underwent unilateral Vim‐FUS or STN‐FUS between June 2015 and August 2022. Patients scored ≥2 for postural and/or resting tremor on the most affected body side in the off‐medication state. The primary outcome was the between‐group difference in tremor improvement on the treated side at 12‐month follow‐up, including a responder's analysis. Data regarding safety, global motor status, and dopaminergic requirements were also collected. Group comparisons used repeated measures ANOVA with Bonferroni correction; statistical significance for *P* < 0.05.

**Results:**

Among 175 patients treated at the two sites, 63 were included (23 Vim‐FUS, 40 STN‐FUS). At baseline, both groups were equivalent in disease duration (6.7 ± 3.8 vs. 6.1 ± 3.4 years, *P* = 0.48) and tremor severity (5.7 ± 1.5 vs. 5.9 ± 2.5, *P* = 0.7). While the benefit in tremor was equivalent between the groups at 4 months (*P* = 0.15), tremor reduction was greater in STN‐ FUS patients at 12 months (4.4 ± 2.0, 95% CI 3.7–5.0 compared with 2.7 ± 3.7, 95% CI 1.1–4.3 for Vim‐FUS, *P* = 0.012). In 47.5% (19/40) of STN‐FUS patients tremor was completely abolished versus 8.7% (2/23) in Vim‐FUS patients (*P* < 0.01). Most adverse events were mild (91%) and resolved by 12 months.

**Conclusions:**

STN‐FUS and Vim‐FUS significantly improved medication‐refractory PD tremor; however, subthalamotomy might have greater and more sustained effect. © 2025 The Author(s). *Movement Disorders* published by Wiley Periodicals LLC on behalf of International Parkinson and Movement Disorder Society.

Tremor is frequent in people with Parkinson's disease (PD) and significantly impairs their quality of life and daily activities.^1^ Medical therapy (ie, antiparkinsonian drugs) often has a limited effect.^2^ In medication‐refractory tremor, invasive treatments such as deep brain stimulation (DBS) and, more recently, MRI‐guided high‐intensity focused ultrasound (FUS) incisionless ablation may be considered.^3^ The ventral intermediate nucleus of the thalamus (Vim) is the classically acknowledged neurosurgical target for treating refractory tremor, including that associated with PD.^4^ Indeed, targeting the thalamus was the initial approach of DBS to treat parkinsonian tremor.^5^ However, the limited effect on other parkinsonian features like bradykinesia and rigidity led to the subthalamic nucleus (STN) being chosen as the main target of stimulation for PD due to its ability to improve the whole spectrum of motor features, including tremor.^6^, ^7^

In the case of FUS ablation, the thalamus has also been the initially favored target to treat parkinsonian tremor. Unilateral lesioning of the thalamic Vim (ie, thalamotomy) with FUS (Vim‐FUS) has been shown to significantly ameliorate PD tremor in a randomized sham‐controlled trial.^8^ Nevertheless, the 12‐month follow‐up of the study suggested a partial loss of benefit over time. This raises the question of whether alternative brain targets for ultrasound ablation might offer sustained efficacy for medication‐refractory parkinsonian tremor, as observed with DBS. To date, several studies have explored the efficacy of FUS to address motor manifestations of PD by targeting the subthalamic nucleus (STN‐FUS),^9^ the globus pallidus internus (GPi‐FUS),^10^ and the pallido‐thalamic tract (PTT‐FUS).^11^ Long‐term follow‐up studies are only available for Vim‐FUS and STN‐FUS. Importantly, while the former show mixed outcomes in terms of benefit persistence,^8^, ^12^, ^13^, ^14^, ^15^ STN‐FUS evidence suggests sustained improvement in parkinsonian motor features, encompassing tremor.^16^ However, formal comparisons between target long‐term efficacy are lacking. Consequently, relevant points such as the persistence (or lack) of the initial benefit, and the impact against bradykinesia and rigidity, are generally not considered when deciding the treatment target for a given PD patient with medically‐refractory tremor. Here, we report a two‐center, retrospective study aiming to compare the effect of unilateral Vim‐FUS and STN‐FUS on patients with medication‐refractory PD tremor.

## 1 Methods

### 1.1 Participants and Study Design

PD patients suffering from medically‐refractory tremor who underwent FUS ablation targeting the Vim or the STN in Kiel and Madrid between June 2015 and August 2022 were identified in the databases. They were considered for analysis if they fulfilled the following criteria: (1) PD diagnosis according to the Movement Disorder Society criteria^17^; (2) treatment with Vim‐FUS or STN‐FUS; (3) presence before treatment of upper or lower limb resting or postural tremor with severity of ≥2 in at least one of the lateralized tremor items on the side of the body to be treated, according to the Motor part of the Movement Disorder Society‐Unified Parkinson's Disease Rating Scale‐Part III (MDS‐UPDRS‐III),^18^ which has been demonstrated to be valid and reliable for independently assessing tremor in PD^19^; and (4) standardized clinical assessment performed at baseline, 4 (±1.5 months), and 12 months (±1 month) after treatment. Data were collected prospectively and encompassed baseline demographics, dopaminergic therapy (levodopa equivalent daily dose, LEDD),^20^ the MDS‐UPDRS‐Parts II‐IV, and treatment‐related adverse events. Exclusion criteria were: (1) tremor due to other concomitant neurological pathology or acquired etiology, (2) prior brain surgery including DBS, (3) missing data, or (4) dropout before 12 months. The study was approved by the local ethics committees (Kiel University: D605/20 and D606/20; Madrid CEIm: 24.06.2358‐GHM). Patients signed informed consent before receiving treatment. The Strengthening the Reporting of Observational Studies in Epidemiology (STROBE) guidelines have been followed.^21^

### 1.2 Outcomes

#### 1.2.1 Primary Endpoints

The primary endpoint was the between‐group difference in tremor reduction on the treated body side in the off‐medication state (ie, after a minimum of 12 h of withdrawal of dopaminergic medication, thus reflecting the net effect of ablation) throughout 12 months of follow‐up. Tremor was assessed using the sum of the MDS‐UPDRS unilateral items 3.15 (postural upper limb tremor), 3.16 (upper limb kinetic tremor), and 3.17 (upper and lower limb resting tremor), range 0–16 points, with higher scores indicating more severe tremor. To measure therapy outcome on an individual basis, a treatment‐responders analysis was also conducted. A severity measure for tremor on the treated body side was applied using the maximum score of the four tremor amplitudes (rest, postural, and kinetic upper and lower limb tremor), ie, a number between 0 and 4. The MDS‐UPDRS tremor scores were used as anchors: none (0), slight (1, ≤1 cm), mild (2, 1–3 cm), moderate (3, 3–10 cm), and severe (4, >10 cm). Patients who scored 0 after treatment were defined as full responders, those scoring 1 as partial responders, those scoring 2 as insufficient responders, and patients with scores of 3 or higher as non‐responders.

#### 1.2.2 Secondary Endpoints

Secondary endpoints included the between‐ and intra‐group differences in improvement in bradykinesia and rigidity on the treated body side (MDS‐UPDRS items 3.4–3.8; range 0–20 points, and items 3.3.2–3.3.5; range 0–8 points, respectively), the response of all motor signs on the treated side (sum of unilateral rigidity, bradykinesia, and tremor; range 0–44 points), the global motor status according to the MDS‐UPDRS‐Part III (range 0–132 points), and the severity of motor complications (MDS‐UPDRS‐Part IV, range 0–24 points) with higher scores indicating higher severity in all cases. All motor assessments were performed in the off‐medication state. Additional clinical outcomes included independence in activities of daily living (MDS‐UPDRS‐Part II, range 0–52, higher scores indicating higher disability) and changes in LEDD. The registry of treatment‐related adverse events included the nature of each adverse event, date of onset, outcome, and action taken. Their severity (ie, mild, moderate, or severe) was rated by the treating physician according to the US Food and Drug Administration's definition of adverse events and their functional impact. Thus, signs or symptoms with no or minimal interference with daily activities were considered mild; moderate indicated symptoms that cause greater than minimal interference in functional activities, with noninvasive intervention indicated; and severe indicated symptoms that disrupt activities of daily living, with intervention or hospitalization indicated. Procedure details and lesion location and size in follow‐up brain MRI were also compared.

### 1.3 Treatment Details and Lesion Topography Analysis

Target selection criteria were similar in both sites and the decision was based on clinical presentation. Thus, patients with high predominance of tremor over the other cardinal motor features received Vim‐FUS, whereas the presence of meaningful bradykinesia/rigidity led to consideration of the STN. Also, frailer patients or those presenting cognitive impairment who were less likely to tolerate a long procedure were favored for the usually shorter Vim‐FUS. The general approach for both targets is a standard procedure and has been developed jointly by both teams, which performed thalamotomy and subthalamotomy with the same methodology. This includes patient preparation, calculation of the skull density ratio (ie, a measure of ultrasound energy penetration through the skull), stereotactic starting coordinates, intraprocedural movements, number of effective sonications (ie, energy delivery reaching the >54°C threshold of permanent ablation), and topography. Post‐treatment ablation size and location of lesion centroids were studied after manual segmentation on T1‐w MRI acquired 24 h after the procedure. See Data S1 for further procedure information.

### 1.4 Statistical Analysis

The normal distribution of data was controlled using the Shapiro–Wilk test. The independent *t*‐test or the equivalent nonparametric test was used for between‐group baseline comparison where appropriate. The group comparisons were performed using repeated‐measure ANOVA (for analysis of differences between means at baseline, 4‐month, and 12‐month visits) or ANOVA (comparison of mean treatment deltas at each timepoint), and post hoc testing. The assumptions for repeated measure ANOVA and ANOVA were controlled and, if violated, a Greenhouse–Geisser correction or Kruskal–Wallis test was applied. *P*‐values were adjusted by Bonferroni correction. Omega squared (ꙍ2) effect size calculation was used to provide an unbiased effect size measure. The percentage change from baseline was calculated as follows: (baseline scores minus 4‐month and 12‐month scores/baseline scores) × 100. For the responder analysis, the Fisher exact test was used. Correlation analysis and multiple linear regression were performed to account for the influence of variables showing baseline statistically significant differences between groups. Adverse events are presented descriptively. Statistical analysis was performed using JASP Team (version 0.17, University of Amsterdam, The Netherlands). *P* < 0.05 values were considered statistically significant.

## 2 Results

**FIG. 1:**
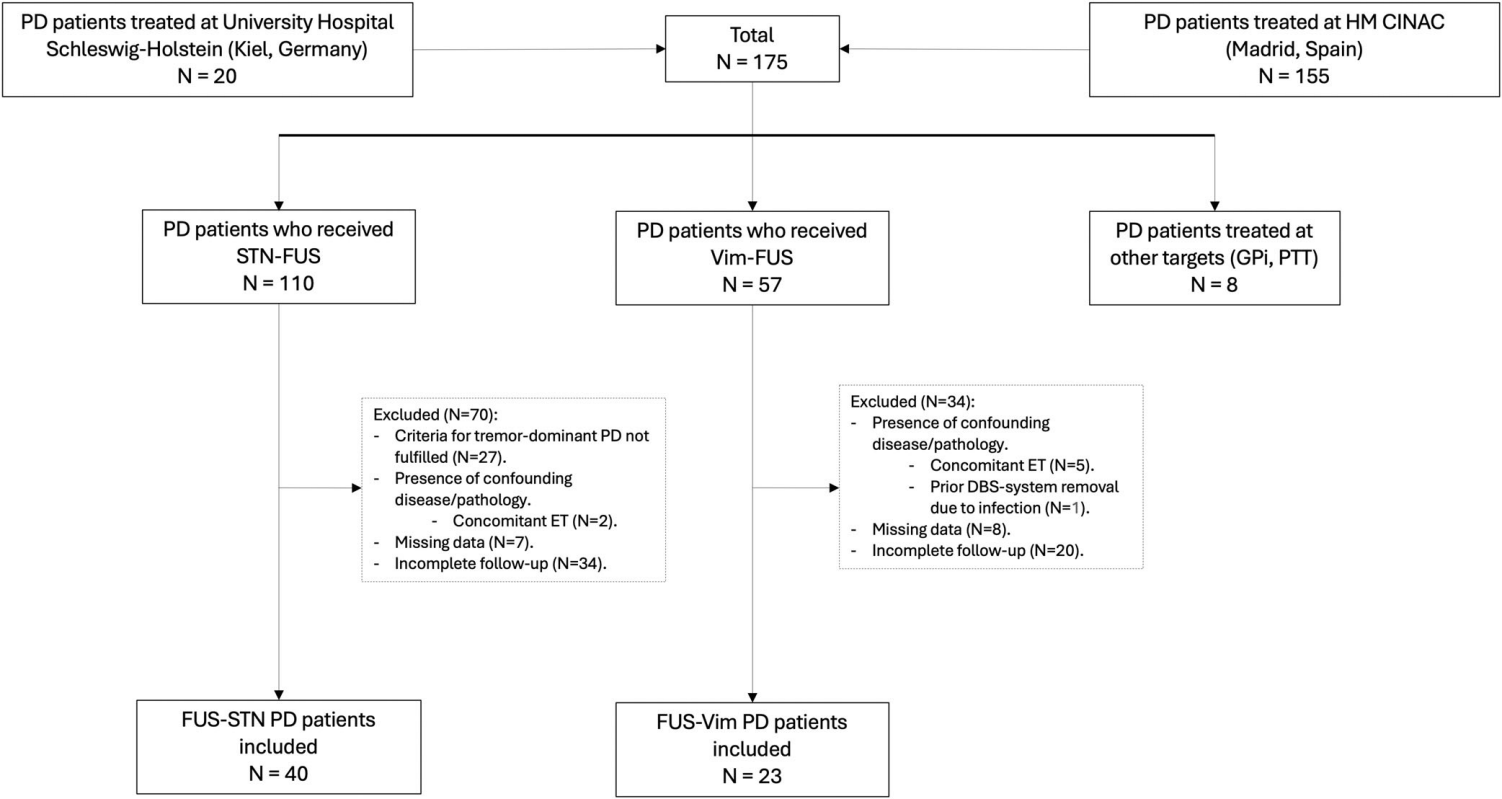
Patient flowchart. PD, Parkinson's disease; STN‐FUS, ablation of the subthalamic nucleus; Vim‐FUS, ablation of the ventral‐intermediate nucleus; GPI, globus pallidus internus; PTT, pallido‐thalamic tract; ET, essential tremor; DBS, deep brain stimulation.

A total of 175 PD patients received FUS ablation: 155 from Madrid and 20 from Kiel. Of these, 110 patients received STN‐FUS and 57 Vim‐FUS. The remaining 8 subjects were treated in other targets (GPi‐FUS and PTT‐FUS). Sixty‐three patients met the inclusion/exclusion criteria: 40 underwent STN‐FUS and 23 Vim‐FUS (flowchart in Fig. 1).

At baseline, STN‐FUS‐treated patients were significantly younger compared with Vim‐FUS patients (58.6 ± 9.8 years vs. 70.8 ± 8.9 years, respectively, *P* < 0.001) (Table 1). Mean disease duration was comparable between the two groups (STN‐FUS: 6.1 ± 3.4 years, Vim‐FUS: 6.7 ± 3.8 years, *P* = 0.48). Tremor severity on the side to be treated was equivalent between groups (mean score 5.7 ± 1.5 for the STN‐FUS group, and 5.9 ± 2.5 for the Vim‐FUS group, *P* = 0.7). Conversely, patients treated with STN‐FUS demonstrated significantly higher scores for bradykinesia (STN‐FUS: 10.1 ± 2.5 vs. Vim‐FUS: 8.3 ± 2.0, *P* = 0.004) and a higher MDS‐UPDRS‐IV score (STN‐FUS: 3.3 ± 3.9 vs. Vim‐FUS: 0.48 ± 1.1, *P* < 0.001, Fig. S3). Motor impairment on the to‐be‐treated body side was higher in the STN‐FUS group, although non‐significantly (STN‐FUS: 19.1 ± 3.4 vs. Vim‐FUS: 17.4 ± 3.7, *P* = 0.07). The MDS‐UPDRS‐III score did not differ significantly between the two groups (STN‐FUS: 37.9 ± 9.7 vs. Vim‐FUS: 40.3 ± 10.6, *P* = 0.37), nor did dopaminergic drug requirements (STN‐FUS: 717.1 ± 299.1 mg vs. Vim‐FUS: 620.2 ± 396.3 mg, *P* = 0.27).

**TABLE 1:** Patient baseline characteristics

Characteristic	Vim‐FUS		STN‐FUS		*P‐*value
Patients (n)	23		40		
Gender, male	16/23	70%	25/40	63%	
Age (years)	70.8	(±8.9)	58.6	(±9.8)	<0.001***
Disease duration (years)	6.7	(±3.8)	6.1	(±3.4)	0.48
Tremor t.s.^a^ (MDS‐UPDRS 3.15–3.17.4)	5.9	(±2.5)	5.7	(±1.5)	0.7
Bradykinesia t.s.^a^ (MDS‐UPDRS 3.4–3.8)	8.3	(±2.0)	10.1	(±2.5)	0.004**
Rigidity t.s.^a^ (MDS‐UPDRS 3.3.2–3.3.5)	3.3	(±1.4)	3.3	(±0.9)	0.34
Motor signs t.s.^a^ (MDS‐UPDRS 3.3.2–3.82 + 3.15–3.17.4)	17.4	(±3.7)	19.1	(±3.4)	0.07
MDS‐UPDRS‐Part III (total)^a^	40.3	(±10.6)	37.9	(±9.7)	0.37
MDS‐UPDRS‐Part II	10.1	(±5.5)	10.1	(±6.0)	0.97
MDS‐UPDRS‐Part IV	0.48	(±1.1)	3.3	(±3.9)	<0.001***
Total LEDD (mg/day)	620.2	(±396.3)	717.1	(±299.1)	0.27

*Note*: Data are given as mean (±SD).

* *P* < 0.05.

** *P* < 0.01.

*** *P* < 0.001.

^a^ MDS‐UPDRS‐III assessments were considered in the off‐medication state.

Abbreviations: Vim‐FUS, ablation of the ventral‐intermediate nucleus; STN‐FUS, ablation of the subthalamic nucleus; t.s., treated body side; MDS‐UPDRS, Movement Disorder Society‐Unified Parkinson Disease Rating Scale; LEDD, levodopa equivalent daily dose.

**FIG. 2:**
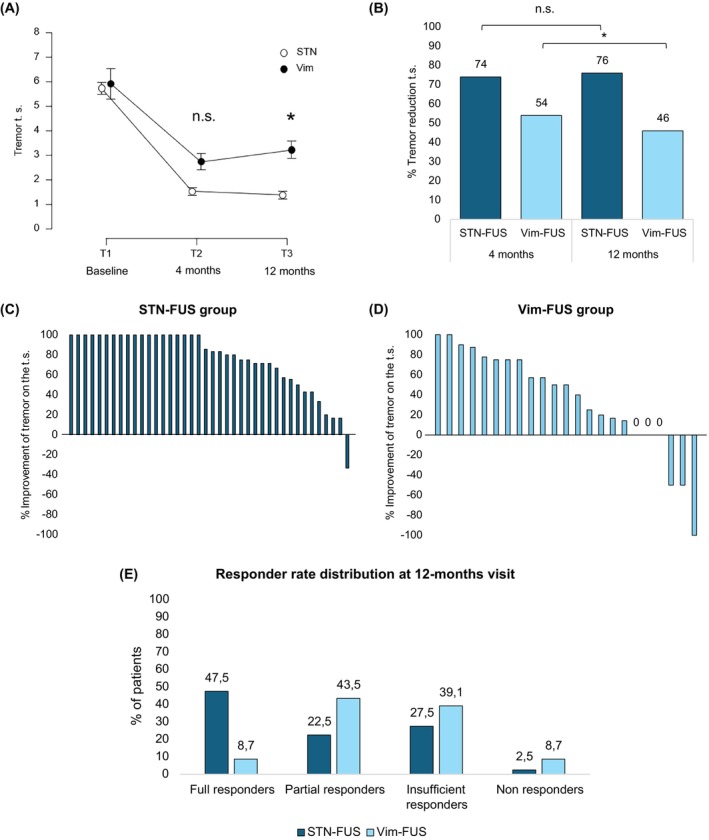
Effect of focused ultrasound thalamotomy and subthalamotomy on parkinsonian tremor. Shown are the absolute tremor reduction (A), percentage of tremor reduction (B), and the individual responses to ablation of the subthalamic nucleus (STN‐FUS) (C) and ablation of the ventral‐intermediate nucleus (Vim‐FUS) (D) on the treated side of the body at 12 months. Tremor raw scores and percentage of change are assessed according to the Movement Disorder Society‐Unified Parkinson's Disease Rating Scale‐Part III (MDS‐UPDRS‐III) tremor subscore after 12 h of dopaminergic drug withdrawal (off‐state). Both treatments improved tremor significantly but, at 12 months, the benefit of STN‐FUS over tremor was superior to Vim‐FUS, as patients treated with the latter showed a decrease of benefit from 4 to 12 months. (E) shows responder rate distribution at 12 months for both STN‐FUS and Vim‐FUS patients. Responders were defined depending on tremor score at 12 months. Patients scoring 0 were defined as full responders, those scoring 1 as partial responders, those scoring 2 as insufficient responders, and patients with scores of 3 or higher as non‐responders. Data are given as mean ± SE. *P < 0.05. Follow‐up timepoints windows correspond to 4 (±1.5) months and 12 (±1) months after unilateral focused ultrasound ablation. STN‐FUS, ablation of the subthalamic nucleus; Vim‐FUS, ablation of the ventral‐intermediate nucleus; n.s., not significant; t.s., treated side. [Color figure can be viewed at wileyonlinelibrary.com]

Both groups had significant tremor improvement on the treated side compared with baseline at both 4‐ and 12‐month time points (all *P* < 0.001). However, while at 4 months tremor reduction was not significantly different between targets (Δ4.2 ± 2.0 points, 95% CI 3.6–4.8, in STN‐FUS patients vs. Δ3.2 ± 3.7 points, 1.6–4.8, in the Vim‐FUS group, *P* = 0.15), at 12 months, the tremor reduction from baseline was greater in STN‐FUS patients with 4.4 ± 2.0 points (3.7–5.0, 76%) compared with 2.7 ± 3.7 points (1.1–4.3, 46%) in the Vim‐FUS group (*P* = 0.012). Details are provided in Figure 2 and Table 2. The responder analysis showed that 12 months after the procedure, 47.5% of patients from the STN‐FUS group (n = 19/40) were full responders (ie, scored 0) compared with only 8.7% (2/23) in the Vim‐FUS group (*P* = 0.002; Figs. 2 and S1). Analysis of the effects on resting, postural, and intention tremor showed a significantly greater effect of STN‐FUS than Vim‐FUS at both 4 and 12 months. At 12 months, the difference between STN‐FUS and Vim‐FUS was greatest for resting tremor (69% reduction vs. 31% reduction, *P* < 0.001), followed by kinetic tremor (81% vs. 49%, *P* < 0.001) and postural tremor (83% vs. 63%, *P* < 0.001). Correlation and multiple linear regression analysis of treatment effects on tremor were unrelated to age.

**TABLE 2:** Primary and secondary outcomes

Timepoint	Baseline		4 months					12 months				
Target	Vim‐FUS	STN‐FUS	Vim‐FUS	STN‐FUS	Vim Δ from baseline	STN Δ from baseline	Difference between Δ, *P*‐value	Vim‐FUS	STN‐FUS	Vim‐FUS Δ from baseline	STN‐FUS Δ from baseline	Difference between Δ, *P*‐value
Primary outcome												
Tremor t.s.^a^ (MDS‐UPDRS 3.15–3.17.4)	5.9 (±2.5)	5.7 (±1.5)	2.7 (±2.5)	1.5 (±1.7)	3.2 (±3.7)	4.2 (±2.0)	0.15	3.2 (±2.3)	1.4 (±1.7)	2.7 (±3.7)	4.3 (±2.0)	0.012*
Secondary outcomes												
Bradykinesia t.s.^a^ (MDS‐UPDRS 3.4–3.8)	8.3 (±2.0)	10.1 (±2.5)	5.6 (±2.3)	4.9 (±2.8)	2.7 (±3.6)	5.2 (±2.4)	0.001**	6.0 (±2.2)	5.1 (±2.6)	2.3 (±3.6)	5.0 (±2.5)	<0.001***
Rigidity t.s.^a^ (MDS‐UPDRS 3.3.2–3.3.5)	3.3 (±1.4)	3.3 (±0.9)	1.7 (±1.5)	1.4 (±1.2)	1.6 (±1.6)	2.0 (±1.0)	0.3	1.6 (±1.3)	1.5 (±1.1)	1.7 (±1.8)	1.8 (±1.1)	0.72
Motor signs t.s.^a^ (MDS‐UPDRS 3.3.2–3.82 + 3.15–3.17.4)	17.4 (±3.7)	19.1 (±3.4)	10.0 (±4.7)	7.8 (±4.3)	7.4 (±6.5)	11.4 (±3.9)	0.021*	10.8 (±4.6)	8.0 (±4.2)	6.7 (±6.7)	11.1 (±3.6)	0.01*
MDS‐UPDRS‐Part III^a^	40.3 (±10.6)	37.9 (±9.7)	29.4 (±9.0)	22.6 (±11.0)	11.0 (±10.3)	15.4 (±5.6)	0.031*	30.1 (±9.1)	23.0 (±10.0)	10.2 (±10.7)	15.0 (±5.9)	0.024*
MDS‐UPDRS‐Part II	10.1 (±5.5)	10.1 (±6.0)	7.7 (±5.6)	8.0 (±6.5)	1.6 (±2.5)	2.1 (±5.3)	0.65	7.7 (±4.5)	7.9 (±7.3)	1.6 (±2.6)	2.2 (±4.7)	0.54
MDS‐UPDRS‐Part IV	0.48 (±1.1)	3.3 (±3.9)	0.83 (±2.4)	2.8 (±3.2)	−0.35 (±2.7)	0.54 (±3.1)	0.26	0.35 (±0.65)	3.0 (±3.8)	0.13 (±1.3)	0.33 (±3.4)	0.79
Total LEDD (mg/day)	620.2 (±396.3)	717.1 (±299.1)	588.2 (±367.6)	611.1 (±301.3)	32.0 (±132.2)	105.9 (±176.4)	0.086	617.4 (±360.6)	675.3 (±292.6)	2.7 (±138.9)	41.7 (±216.2)	0.44

*Note*: Data are presented as mean (±SD). For all outcomes, positive values indicate reduction, ie, improvement.

* *P* < 0.05.

** *P* < 0.01.

*** *P* < 0.001.

^a^ MDS‐UPDRS‐III assessments were considered in the off‐medication state.

Abbreviations: LEDD, levodopa equivalent daily dose; MDS‐UPDRS, Movement Disorder Society‐Unified Parkinson Disease Rating Scale; STN‐FUS, ablation of the subthalamic nucleus; t.s., treated body side; Vim‐FUS, ablation of the ventral‐intermediate nucleus.

STN‐FUS reduced bradykinesia and overall motor features on the treated side more effectively. At the 12‐month visit, bradykinesia showed a reduction of 5.0 ± 2.5 points (4.1–5.7, 49%) versus 2.3 ± 3.6 points (0.7–3.8, 27%) for Vim‐FUS (*P* < 0.001). The treated side was reduced by 11.1 ± 3.6 points (9.7–12.2, 58%) in the STN‐FUS group versus 6.7 ± 6.7 points (3.8–9.6, 38%) in Vim‐FUS patients (*P* = 0.01). A similar effect was detectable for the MDS‐UPDRS‐III (*P* = 0.024). Post hoc testing showed a reduction from baseline of 15 ± 5.9 points (13.1–16.9, 40%) by STN‐FUS and 10.2 ± 10.7 points (5.6–14.8, 25%) by Vim‐FUS. Rigidity on the treated side, MDS‐UPDRS‐II, MDS‐UPDRS‐IV, and total LEDD were reduced equally by both STN‐FUS and Vim‐FUS (Table 2, Fig. S2).

The most frequent post‐procedural adverse event was gait imbalance (53% of STN‐FUS patients and 70% of Vim‐FUS patients). In the STN‐FUS cohort, all cases were mild, whereas 13% of Vim‐FUS patients experienced moderate imbalance, with only one Vim‐FUS patient still mildly affected at 12 months. Off‐medication dyskinesia occurred only in the STN‐FUS group (15%) during the immediate post‐procedural period. New‐onset levodopa‐induced dyskinesia (ie, on‐medication) occurred within the first days post‐procedure more frequently after STN‐FUS (27.5%) compared with Vim‐FUS (4%). Most cases were mild, except for two moderate STN‐FUS cases. Dyskinesia improved after adjustment of medication and, at 12 months, persisted mildly while on‐medication in 3 STN‐FUS patients (8%) versus none in the Vim‐FUS group. Sensory disturbances (finger and oral paresthesia) were predominant in the Vim‐FUS group (22% and 17%, respectively versus 3% and none in STN‐FUS patients). All were mild except for one Vim‐FUS moderate case, and, by 12 months, 4 Vim‐FUS patients (17%) still reported mild sensory disturbances. Post‐procedural speech disturbances were more frequent among STN‐FUS patients (48% vs. 22%); all cases were mild and persisted by 12 months in only 3% of STN‐FUS patients versus 9% in Vim‐FUS. Contralateral motor weakness occurred in 15% of STN‐FUS and 13% of Vim‐FUS subjects. Most patients fully recovered but one case per group reported difficulties while performing fine motor tasks 12 months after treatment. Post‐treatment, 18% of STN‐FUS patients experienced progressive weight gain, decreasing to 10% at 12 months. Three cases (8%) of mild behavioral disinhibition were reported during the first weeks after STN‐FUS, spontaneously resolving within a few weeks. Correlation and multiple linear regression analysis showed that the number of adverse events was not related to age. Details on adverse events can be found in Table 3.

**TABLE 3:** Adverse events

	Immediately post‐procedure n (%)		4 months n (%)		12 months n (%)	
Adverse event	Vim‐FUS	STN‐FUS	Vim‐FUS	STN‐FUS	Vim‐FUS	STN‐FUS
Imbalance	16 (70)	21 (53)	4 (17)	2 (5)	1 (4)	0
Off‐med dyskinesia^a^	0	6 (15)	0	0	0	0
On‐med dyskinesia	1 (4)	11 (27.5)	1 (4)	11 (27.5)	0	3 (8)
Dysarthria	5 (22)	19 (48)	2 (9)	4 (10)	2 (9)	1 (3)
Facial asymmetry	1 (4)	7 (18)	1 (4)	2 (5)	1 (4)	1 (3)
Contralateral limb weakness	3 (13)	6 (15)	1 (4)	3 (8)	1 (4)	1 (3)
Weight gain	0	0	0	7 (18)	0	4 (10)
Cheerfulness/disinhibition	0	3 (8)	0	1 (3)	0	0
Limb dystonia	0	0	3 (13)	2 (5)	2 (9)	0
Dysmetria	1 (4)	2 (5)	1 (4)	0	0	0
Finger paresthesia	5 (22)	1 (3)	5 (22)	1 (3)	2 (9)	0
Oral paresthesia	4 (17)	0	3 (13)	0	2 (9)	0
Dysgeusia	4 (17)	1 (3)	2 (9)	1 (3)	1 (4)	0
Adverse events: severity and number of events (n)						
Mild	37	79	19	30	11	9
Moderate	3^b^	4^c^	4^d^	3^e^	1^g^	1^h^
Severe	0	0	0	1^f^	0	0

*Note*: Adverse events occurrence in the upper rows are presented as number of patients with percentages in parentheses. The severity in the lower rows is presented as number of adverse events. Severity was categorized as mild, moderate, and severe according to functional impact.

^a^ Patients who experienced off‐medication dyskinesia also had dyskinesia while on‐medication.

^b^ All cases were of imbalance.

^c^ 1 case of new‐onset on‐med dyskinesia, 1 case of limb clumsiness, 1 case of behavioral disinhibition, 1 case of weight gain.

^d^ 1 case of imbalance, 2 cases of foot dystonia, 1 case of oral paresthesia.

^e^ 1 case of new‐onset on‐med dyskinesia, 2 cases of weight gain.

^f^ 1 case of foot dystonia.

^g^ 1 case of oral paresthesia.

^h^ 1 case of weight gain.

Abbreviations: off‐med, off medication; on‐med, on medication; STN‐FUS, focused ultrasound subthalamotomy; Vim‐FUS, focused ultrasound thalamotomy.

STN‐FUS treatments were longer than Vim‐FUS procedures (216.4 ± 79.8 min vs. 140.5 ± 29.2 min, *P* < 0001) and presented a higher number of intraprocedural movements (3.1 ± 0.9 vs. 2.2 ± 0.8, *P* < 0.001) and of sonications (19.9 ± 6.7 vs. 12.6 ± 3.9, *P* < 0.001). The number of sonications exceeding 54°C was significantly higher in the STN‐FUS group (7.8 ± 3.4 vs. 4.6 ± 1.8, *P* < 0.001). STN‐FUS lesions were larger than Vim‐FUS lesions (274.7 ± 135.3 mm^3^ vs. 210.9 ± 98.6 mm^3^, *P* = 0.048). STN‐FUS topography analysis showed an average lesion located more ventral (*Z* = −6.6 ± 1.1 mm vs. *Z* = −1.7 ± 1. mm, *P* < 0.00.1), anterior (*Y* = −15.6 ± 1.2 mm vs. *Y* = −17.2 ± 1.4 mm, *P* < 0.001), and medial (*X* = −12.7 ± 0.7 mm vs. *X* = −14.7 ± 0.7 mm, *P* < 0.001) than Vim‐FUS. See Table S1, Methods S1 and S2, Figure S4 for procedure and lesion topography details.

## 3 Discussion

This two‐center study compared the effect of unilateral Vim‐FUS versus STN‐FUS on medically‐refractory PD tremor. Although both approaches significantly improved tremor, after 12 months tremor control was superior in the STN‐FUS group compared with the Vim‐FUS group. In addition, the percentage of patients reaching total tremor abolishment, was significantly higher for STN‐FUS‐treated subjects (47.5%) than for patients who received Vim‐FUS (8.7%). Bradykinesia on the treated side and total motor score also improved more after STN‐FUS, whereas the effect on rigidity was equivalent between targets. Adverse events were frequent in both groups but mostly mild, and some were practically target‐specific such as dyskinesia in STN‐FUS patients and sensory disturbances in Vim‐FUS‐treated subjects. There was no statistically significant discrepancy at 1 year between the two cohorts in other outcomes.

Previous experience with radiofrequency lesioning and DBS targeting both the STN and the thalamic Vim showed long‐lasting improvements for PD tremor.^7^, ^22^, ^23^, ^24^, ^25^ Despite the experience with stimulation, when FUS was developed, safety concerns regarding subthalamic ablation led to a preference for Vim thalamotomy for treating parkinsonian tremor.^8^, ^26^ Initial studies suggested that Vim‐FUS became less effective over time: the pivotal unilateral Vim‐FUS for PD trial found that initial tremor improvement diminished by 12 months, with reduction of at least 50% being evident only in 55% of cases.^8^ In a subsequent series including 26 patients who were followed for up to 5 years, 10 subjects (38%) showed a loss of efficacy.^15^ Contrarily, the 3‐year follow‐up data of 49 tremor‐dominant PD patients treated with Vim‐FUS has recently reported sustained benefit for 1 and 3 years, although approximately half of the patients were unavailable for evaluation at the 1‐year follow‐up.^14^ Finally, a case series with PD patients, with essential tremor and 24 with dystonic tremor found a decline of Vim‐FUS efficacy only for parkinsonian tremor.^13^ STN‐FUS to treat PD was initially explored in a pilot study including 10 patients and showed remarkable motor improvement, with tremor being the most beneficial.^27^ These results were confirmed by an STN‐FUS double‐blinded randomized controlled trial that demonstrated a 52.6% improvement for the treated body side, and significant improvements of 60%, 33.3%, and 83.3% for rigidity, bradykinesia, and tremor, respectively.^9^ Importantly, the 3‐year follow‐up of these two studies suggested a sustained benefit on tremor.^16^ More recently, a case series study that explored the safety and efficacy of unilateral STN‐FUS in PD patients less than 5 years from diagnosis and a new case series from a different group both showed an improvement of 91% for tremor 1 year after the procedure.^28^, ^29^ Thus, the available evidence for ultrasound ablation is consistent with our current results, suggesting that STN‐FUS might have a more sustained effect on parkinsonian tremor compared with Vim‐FUS.

We observed a significant anti‐rigidity effect from thalamotomy. Despite such an effect being well‐documented in the early functional neurosurgery literature,^30^ it is not part of the current functional neurosurgical understanding. Traditionally, the ventrolateral thalamus anti‐rigidity effect was related to more rostrally placed lesions, the pallidal receiving region, rather than Vim, which relates to proprioceptive afferents and dentate cerebellar output.

Additional outcomes, such as functional disability, motor complications, or dopaminergic drug requirements, were equivalently changed by both Vim‐FUS and STN‐FUS. This can be expected given the net motor benefit provided by both treatments, as indicated by the extensive literature on FUS and DBS.^4^, ^9^, ^15^, ^27^, ^31^, ^32^ Admittedly, changes in dopaminergic drugs were limited, particularly in the Vim‐FUS group.

Our findings also show that adverse events are equally frequent after both STN‐FUS and Vim‐FUS and, in alignment with previous studies,^8^, ^14^, ^16^ most of them were mild and had resolved by the 12‐month follow‐up visit. Notably, the nature of complications varied between the two treatment cohorts due to the specific target and its surrounding structures. Dyskinesias after STN‐FUS are frequent but usually resolve within weeks.^9^, ^16^ One case of dyskinesias after Vim‐FUS was observed in our cohort. The development of involuntary movements after thalamic lesions has been reported,^33^, ^34^ including anecdotal cases following VIM‐FUS^32^; however, the occurrence chorea‐ballism after a thalamic lesion is admittedly very uncommon and physiopathologically intriguing.

Conversely, Vim ablation is more commonly related to sensory disturbances, as the medial lemniscus lies immediately caudal to it. Weakness secondary to spinocortical tract impingement or gait imbalance allegedly caused by impact on the cerebello‐thalamic tract occur, according to our analysis, with the same frequency regardless of the target. Thus, the severity of potential adverse events after treatment might not be a major factor when deciding between the two approaches.

The first and main limitation of our study is its retrospective nature. The number of patients lost to follow‐up is significant. The follow‐up rate was similar in both groups, which, while reassuring regarding their comparability, raises the possibility of a bias towards capturing favorable outcomes. This highlights the need to interpret our findings with caution. A prospective, randomized comparative trial would be ideal, but such a comparative study is not planned in the near future. The decision as to the best treatment for refractory PD tremor using FUS is currently a significant clinical issue, and our findings provide the most meaningful available evidence in this regard. Second, the lack of randomization in our study implies that either target was selected by the treatment teams based on best clinical judgment. Importantly though, the severity of tremor on the treated side (our main objective and primary analysis) was comparable at baseline between groups, thus reducing potential selection bias. Interestingly, our retrospective analysis of baseline data showed that patients selected for STN‐FUS had higher bradykinesia scores and more motor complications. This was not specifically agreed upon in advance but illustrates objectively the clinical considerations that were undertaken to determine the profile of patients who were better candidates for each target. Third, STN‐FUS patients were younger than Vim‐FUS patients at study inclusion. Therefore, the STN‐FUS group could have a better treatment effect and compensate better for adverse events. However, an influence of age on the treatment effect or adverse events was not detectable in our study population for either the STN‐FUS or the Vim‐FUS group. In any case, patient frailty and procedural tolerability are factors which were considered when selecting the most appropriate treatment. Whether this will continue to be the case with more experience remains to be seen. Fourth, not all PD patients treated at our centers were included in the analysis and the number of patients contributed by each site is unbalanced, which might bias the results. In any case, the total sample is higher than most FUS studies and may be considered representative. Fifth, while a notable strength of this study is that it was conducted in two centers experienced in functional neurosurgery following the same technical strategy, extrapolation of our findings to other centers with different approaches and levels of experience might be limited. Also, the distribution of patients included is skewed towards a higher number from the Madrid site. This is caused by a longer experience resulting in a larger database. In any case, the two centers contributed patients to both groups (STN and VIM), and the treatment methodology was common to the two teams. Finally, STN‐FUS was associated with a higher number of sonications and higher ultrasound energy dose, resulting in longer treatment durations and larger ablation sizes when compared with the Vim‐FUS group. This can be expected, as STN‐FUS treatments aim to improve all parkinsonian motor signs, requiring impact on each specific sign's sweet spots as is well‐defined in the DBS literature.^35^ In any case, the thalamotomy sizes in our series were larger than those reported in cases of essential tremor successfully treated with Vim‐FUS.^36^, ^37^, ^38^, ^39^, ^40^ A more comprehensive understanding of optimal ablation topography for parkinsonian tremor control is necessary and may enhance Vim‐FUS outcomes in the future, similar to what occurred with Vim‐DBS.^41^

## 4 Conclusions

Both STN‐FUS and Vim‐FUS improve medication‐refractory parkinsonian tremor significantly. However, targeting the STN might offer a superior and more enduring control over tremor compared with Vim‐FUS, coupled with higher benefit on bradykinesia and global motor status. Although safety profiles differed in nature between these two treatment modalities, most reported events were of mild intensity and transient. Patients’ frailty and tolerability to the procedure are factors to consider when choosing between treatments. Prospective studies are advisable; however, in the meantime, our data should serve to help in deciding which approach could be better considered for each individual PD patient.

## Author Roles

(1) Research Project: A. Conception, B. Organization, C. Execution, D. Data Analysis and Interpretation, E. Magnetic Resonance Imaging (MRI) and Technical Data Analysis; (2) Statistical Analysis: A. Design, B. Execution, C. Review and Critique; (3) Manuscript Preparation: A. Writing of the First Draft, B. Review and Critique; (4) Responsible for the Integrity of the Data and the Accuracy of the Data Analysis.

S.P.: 1A, 1B, 1C, 1D, 2A, 2B, 2C, 3A, 3B, 4.

E.N.‐V.: 1A, 1B, 1C, 1D, 2B, 3A, 3B.

J.A.P.‐P.: 1E, 2B, 3B.

M.d.A.: 1A, 1B, 1C, 1D, 3B.

R.R.‐R.: 1A, 1B, 1C, 1D, 1E, 2B, 3B.

J.H.: 3B.

G.D.: 1A, 1B, 1C, 1D, 3A, 3B.

J.A.O.: 1A, 1B, 1C, 1D, 3A, 3B.

A.‐K.H.: 1A, 1B, 1C, 1D, 3B,

R.M.F.: 1A, 1B, 1C, 1D, 2C, 3A, 3B, 4.

## Financial Disclosures of All Authors (for the Preceding 12 Months)

S.P. reports speaker honoraria from Insightec, AbbVie, Medtronic GmbH and Boston Scientific outside the submitted work and grant/research funding from Deutsche Forschungsgemeinschaft (DFG), Parkinson Fonds Deuschland gGmbH, and UCB Pharma GmbH. E.N.‐V. has received financial support from Zambon, Bial, Esteve, and Insightec to attend scientific meetings and received grants from Sociedad Española de Neurología and Asociación Madrileña de Neurología. J.H. has served as a consultant for Stryker Neurovascular and BALT. He has received reimbursement of travel expenses to attend scientific meetings by Rapid Medical outside the submitted work. J.A.P.‐P. has received speaker honoraria from Insightec, Palex, and Zambon outside the submitted work. M.d.A. has received speaker honoraria from Insightec, Palex, and Boston Scientific and reimbursement of travel expenses to attend scientific conferences from Boston Scientific and Medtronic outside the submitted work. R.R.‐R. has received speaker honoraria from Zambon and Insightec outside the submitted work. J.H. reports no disclosures. G.D. has served as a consultant for Boston Scientific, Insightec, and Functional Neuromodulation. He has received royalties from Thieme publishers, funding from the German Research Council (SFB 1261, T1), and private foundations. J.A.O. has received honoraria for lecturing and reimbursement of travel expenses to attend scientific meetings by Insightec outside the submitted work. A.‐K.H. has received lecture fees from Boston scientific and Insightec outside the submitted work. R.M.‐F. reports speaker honoraria from Insightec, Palex, Esteve, Zambon, and Bial outside the submitted work and a grant/research funding from Instituto de Salud Carlos III, Madrid, Spain for health research projects (PI21 Proyectos de investigacion en salud, AES 2021).

## Supporting information


**Data S1.** Supporting Information.

## Data Availability

The data that support the findings of this study are available on request from the corresponding author. The data are not publicly available due to privacy or ethical restrictions.
