# Supplementary material for: Comparative Study of Focused Ultrasound Unilateral Thalamotomy and Subthalamotomy for Medication‐Refractory Parkinson's Disease Tremor
Source: Mov Disord. 2025 Mar 3;40(5):823–33. doi: 10.1002/mds.30159 (PMC12089906; doi:10.1002/mds.30159)
Supplement: Supplementary file 1 — Data S1. Supporting Information. [file MDS-40-823-s001.docx]

**Supplementary Material**

**Comparative study of focused ultrasound unilateral thalamotomy and subthalamotomy for medication refractory Parkinson’s disease tremor**

Steffen Paschen, MD* ^1^; Elena Natera-Villalba, MD* ^2,3^; Jose A. Pineda-Pardo, PhD ^2,4^; Marta del Álamo, MD ^2,4^; Rafael Rodríguez-Rojas, PhD ^2,4^; Johannes Hensler, PhD ^5^, Günther Deuschl, PhD ^1^, Jose A. Obeso, PhD ^2,4,6^ Ann-Kristin Helmers, MD ^#7^; Raúl Martínez-Fernández, PhD ^#^ ^2,4,6^

*Share first co-author. ^#^Share last co-author.

1. Department of Neurology, University Hospital Schleswig-Holstein, Christian-Albrechts-University, Kiel, Germany.
2. Centro Integral de Neurociencias AC (CINAC). HM Universitario Puerta del Sur. Madrid, Spain.
3. PhD Medicine Program, Universidad Autonoma de Madrid, Madrid, Spain.
4. Instituto de Investigación Sanitaria HM Hospitales. Madrid, Spain.
5. Department of Neuroradiology, University Hospital Schleswig-Holstein, Christian-Albrechts-University, Kiel, Germany.
6. Universidad CEU-San Pablo, Madrid, Spain.
7. Department of Neurosurgery, University Hospital Schleswig-Holstein, Christian-Albrechts-University, Kiel, Germany.

**Index of content**

| Figure S1: Patient distribution for each target at baseline and 12 months after the procedure for each tremor subitem. | Page 3 |
| --- | --- |
| Figure S2: Secondary outcomes. | Page 4 |
| Figure S3: Patient distribution at baseline according to tremor severity (MDS-UPDRS III items) and motor complications (MDS-UPDRS IV) | Page 5 |
| Table S1: Procedure data | Page 6 |
| Methods S1. Procedure methodology | Page 7 |
| Methods S2. Topography lesion analysis | Page 9 |
| Figure S4: Frequency maps of Vim and STN ablations | Page 10 |
| References | Page 11 |

**Figure S1: Patient distribution at baseline and 12 months after the procedure of each tremor subitem.**


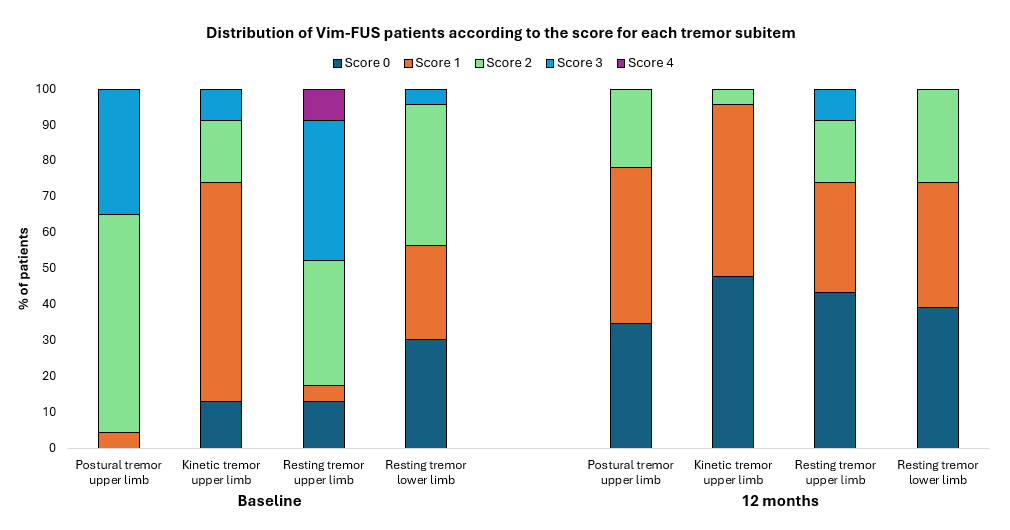


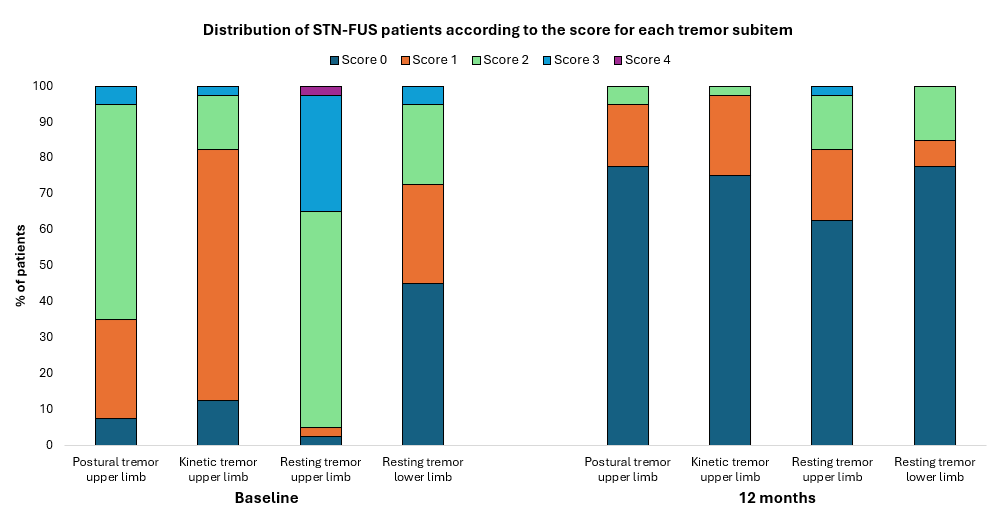


**Figure S2:** **Secondary outcomes.**


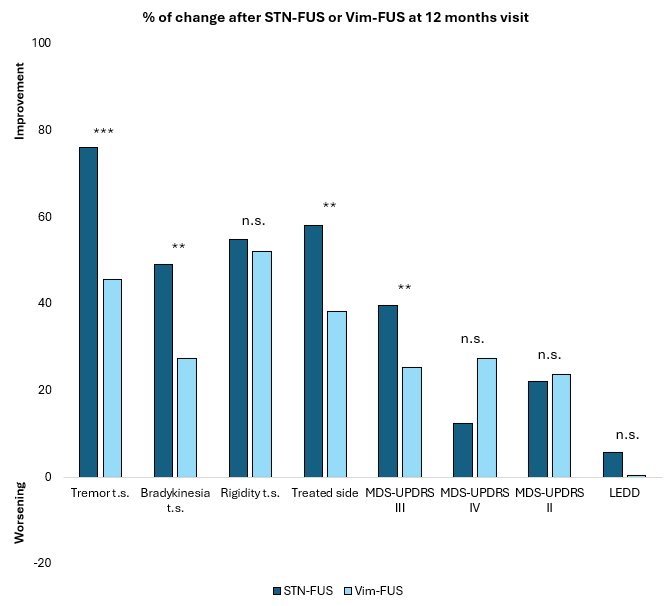


Bradykinesia, motor status of the treated side (treated side total), and general motor status (MDS-UPDRS III) were significantly improved from baseline by STN-FUS compared to Vim-FUS. The change provided by both targets in terms of rigidity, motor complications (MDS-UPDRS IV), and activities of daily living (MDS-UPDRS II) were equivalent. There were no significant differences in LEDD change between the two groups, however, contrary to STN-FUS treated patients, those who received Vim-FUS required increased doses of dopaminergic drugs at 12 months.

Motor assessments were performed in the off-medication state (i.e., at least 12h of dopaminergic drug withdrawal).

Improvement means score reduction for all outcomes except LEDD.

STN-FUS: ablation of the subthalamic nucleus, Vim-FUS: ablation of the ventral-intermediate nucleus, MDS-UPDRS = Movement disorders society Unified Parkinson’s disease rating scale; LEDD = levodopa equivalent daily dose; n.s.=not significant, t. s.=treated body side. Data are given as mean±SE. **P*<0∙05, ***P*<0∙01, ****P*<0∙001.

**Figure S3: Patient distribution at baseline according to tremor severity (MDS-UPDRS III items) and the presence of motor complications (MDS-UPDRS IV).**


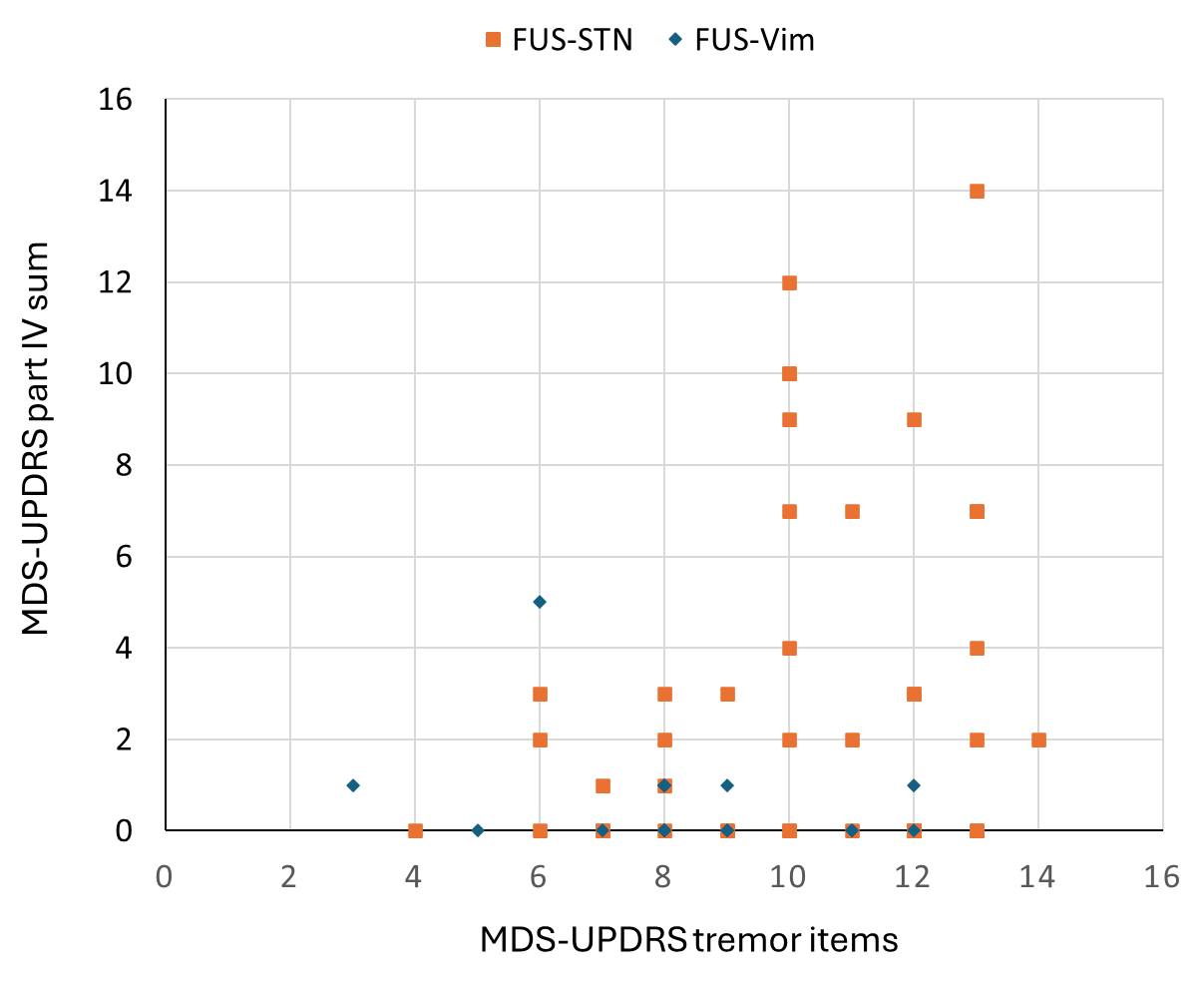


According to the retrospective analysis, the presence and severity of motor complications and tremor were important clinical factors for the choice of treatment target (subthalamotomy versus thalamotomy). Shown are the severity of the motor complications, as assessed with the MDS-UPDRS part IV (y-axis), and the tremor severity (sum of the tremor items of the MDS-UPDRS III, x-axis). With one exception, a subthalamotomy was performed in patients who had moderate to severe motor complications. In patients with mild motor complications, either a subthalamotomy or a thalamotomy was performed.

**Table S1: Procedure data.**

|  | Vim | STN | *P* value |
| --- | --- | --- | --- |
| Skull density ratio | 0∙52 (±0∙1) | 0∙48 (±0∙1) | 0∙18 |
| Skull thickness | 6∙34 (±0∙9) | 6∙54 (±0∙9) | 0∙50 |
| Skull area | 351∙0 | 371∙5 | **0∙006 |
| Number of targets | 2∙2 (±0∙8) | 3∙1 (±0∙9) | ***<0∙001 |
| Number of active elements | 938 ∙0 (±38∙6) | 941∙2 (±39∙2) | 0∙77 |
| Treatment duration (min.) | 140∙5 (±29∙2) | 216∙4 (±79∙8) | ***<0∙001 |
| Sonication duration (min.) | 75∙4 (±25∙8) | 132∙4 (±59∙8) | ***<0∙001 |
| Number of sonications | 12∙6 (±3∙9) | 19∙9 (±6∙7) | ***<0∙001 |
| Number of sonications >52 °C | 6∙2 (±2∙3) | 11∙1 (±4∙3) | ***<0∙001 |
| Number of sonications >54 °C | 4∙6 (±1∙8) | 7∙8 (±3∙4) | ***<0∙001 |
| Number of sonications >57 °C | 2∙2 (±1∙7) | 3∙4 (±2∙2) | *0∙03 |
| Average power (Watts) | 734∙4 (±238∙1) | 799∙4 (±273∙0) | 0∙26 |
| Maximum power (Watts) | 1078∙4 (±258∙9) | 1252∙1 (±390∙0) | *0∙044 |
| Minimal energy (Joules) | 2058∙5 (±833∙4) | 1604∙9 (±423∙8) | *0∙011 |
| Maximum energy (Joules) | 16450∙0 (±9517∙3) | 19256∙1 (±13037 ∙7) | 0∙32 |
| Accumulated energy (Joules) | 115218∙6 (±79231∙2) | 168740∙1 (±76727 ∙7) | **0∙005 |
| Maximum temperature (mean) | 59∙4 (±2∙2) | 60∙3 (±2∙8) | 0∙07 |
| Maximum temperature (max) | 63∙7 (±2∙7) | 65∙1 (±5∙0) | 0∙1 |
| Data are presented as mean (± SD). **P*<0∙05, ***P*<0 ∙01, ****P*<0∙001. | | | |

The mean skull density ratio and mean skull thickness were equivalent between groups (*P*=0∙18 and 0∙50, respectively). The extent of the reduction in tremor severity by Vim-FUS and STN-FUS correlated with the number of sonications (*P*=0∙009), with the average and maximum sonification temperature reached (*P*=0∙004), and the number of active elements (*P*=0∙047).

**Methods S1. Procedure methodology**

General preparations and procedure methodology are equal for both targets and were followed by the two treatment sites. The pPatient’s head is shaved to avoid ultrasound beam distortions resulting from air bubbles stuck in the hair. A stereotactic frame is placed on the patient´s head and subsequently attached to the ultrasound transducer, which is coupled to an MRI machine. The target is defined after obtaining MRI and through a coordinate-based method (see below). Energy delivery (i.e., sonications) is progressively increased to reach ablative temperatures (i.e., above 54ºC). After each sonication, real-time MR thermometry and clinical evaluation is used to ensure proper target location or allow any necessary adjustments. During the procedure, T2-weighted fast spin echo or STIR (for STN-FUS and Vim-FUS, respectively) axial and coronal MRI sequences are performed to verify the location and extent of edema and ablation. The procedure is generally finished once significant clinical improvement and effective impact on target in terms of energy/temperature are achieved.

Initial coordinates for Vim thalamotomy, were established dorso-ventrally at the AC-PC plane, 14 mm lateral to the AC-PC line and 25% anterior from the posterior commissure. Coordinates were further adjusted to the width of the third ventricle and specific patient anatomy. During the treatment, the initial target could be modified depending on the clinical response (presentation of either benefit or adverse events). The general protocol included at least 2 ablative sonications (>54ºC) at the initial target plus enlarging ablation with an additional movement 0.8-1mm mm ventral and posterior to impact the cerebello-thalamic tract that has been associated with a higher tremor benefit ^1,2^. In the second location, one effective sonication was considered sufficient in case tremor was already controlled by that stage.

Initial coordinates to target the subthalamic nucleus were set dorso-ventrally 3mm below the AC-PC plane, 11∙5 to 12 millimeters lateral to AC-PC line, and 2∙5 to 3 millimeters posterior to the mid-commissural point. Coordinates were further adjusted to the width of the third ventricle and specific patient anatomy. The general strategy at both sites consisted of reaching at least two ablative sonications on the initial target, which is known to be the subthalamic motor subregion connected to the supplementary motor area and to have a greater effect on bradykinesia ^3^. Subsequently, a posterolateral movement of 1.5x1.5mm from the first target (adjusted according to patient anatomy and ongoing clinical effect) was performed to impact the subthalamic region connected to cortical M1 that provides higher impact on tremor ^3^. Finally, A third mediodorsal lesion (1.5 mm dorsal, 0·5 mm medial and 1.0 mm anterior from the first target) was performed to impact the pallidothalamic tract, in order to minimize the risk of hemichorea-ballism. ^4,5^

**Methods S2. Topography lesion analysis**

At 24 hours post-procedure, an MRI was acquired to confirm lesion placement. The MRI protocol included T1-weighted (T1-w), T2-weighted (T2-w), and susceptibility-weighted imaging (SWI) images. Lesion boundaries were defined on T1-w images using ITK-Snap segmentation software by identifying the lesion core and periphery, i.e., zones 1 and 2 as described by Wintermark et al.^6^ All images were segmented by at least two independent raters, and only voxels labeled as lesions by both raters were included in the final segmentation.

Baseline T1-w images were pre-processed using the FMRIB Software Library v6.0 (FSL) and Advanced Normalization Tools (ANTs) ^7^ software libraries, along with in-house scripts developed in MATLAB (MathWorks Inc, USA, R2018b). T1-w MRI images were corrected for intensity bias using the N4 bias correction algorithm ^8^, denoised using an optimized non-local means filter ^9^, and skull-stripped using ANTs and skull-stripped using antsBrainExtraction.sh v2.1.0 (using the OASIS template). Skull-stripped T1-w images were co-registered using affine transformation to an MNI ICBM non-linear symmetric T1-w template (mni_icbm152_nlin_sym_09b) and normalized using the non-linear symmetric registration algorithm “SyN” available in ANTs. All lesion masks were normalized to MNI space to create probabilistic representations of the lesions for visual purposes. Topographic measures included lesion volume in native T1-w and standard MNI spaces, as well as centroid coordinates in MNI space.

All metrics, including lesion volume (mm³) and lesion centroid coordinates in MNI ICBM space (mm), were compared between groups using a two-sample unpaired *t*-test. In addition to *P*-values, Cohen d values were reported.

**Figure S4: Frequency maps of Vim and STN ablations.**

Coronal image from the Big Brain template (500 µm isotropic resolution), overlaid with two lesion frequency maps. The FUS-Vim lesion frequency map is displayed in a blue to green color map, and the FUS-STN lesion frequency map in a red to yellow color map. Horizontal black lines indicate the Z coordinate of the average centroid of the lesions across subjects, i.e. Z = -2 mm and Z = -7 mm. On the right **two** axial slices represent the corresponding axial images to these Z-axis locations.

**References:**

1. Pineda-Pardo JA, Martínez-Fernández R, Rodríguez-Rojas R, et al. Microstructural changes of the dentato-rubro-thalamic tract after transcranial MR guided focused ultrasound ablation of the posteroventral VIM in essential tremor. *Hum Brain Mapp*. 2019;40(10):2933-2942. doi:10.1002/hbm.24569
2. Boutet A, Ranjan M, Zhong J, et al. Focused ultrasound thalamotomy location determines clinical benefits in patients with essential tremor. *Brain*. 2018;141(12):3405-3414. doi:10.1093/brain/awy278
3. Rodriguez-Rojas R, Pineda-Pardo JA, Mañez-Miro J, et al. Functional Topography of the Human Subthalamic Nucleus: Relevance for Subthalamotomy in Parkinson's Disease. *Mov Disord*. 2022;37(2):279-290. doi:10.1002/mds.28862
4. Rodriguez-Rojas R, Carballo-Barreda M, Alvarez L, et al. Subthalamotomy for Parkinson's disease: clinical outcome and topography of lesions. *J Neurol Neurosurg Psychiatry*. 2018;89(6):572-578. doi:10.1136/jnnp-2017-316241
5. Lozano AM. The subthalamic nucleus: myth and opportunities. *Mov Disord*. 2001;16(2):183-184. doi:10.1002/mds.1076
6. Wintermark M, Druzgal J, Huss DS, et al. Imaging findings in MR imaging-guided focused ultrasound treatment for patients with essential tremor. *AJNR Am J Neuroradiol*. 2014;35(5):891-896. doi:10.3174/ajnr.A3808
7. Avants BB, Epstein CL, Grossman M, Gee JC. Symmetric diffeomorphic image registration with cross-correlation: evaluating automated labeling of elderly and neurodegenerative brain. *Med Image Anal*. 2008;12(1):26-41. doi:10.1016/j.media.2007.06.004
8. Tustison NJ, Avants BB, Cook PA, et al. N4ITK: improved N3 bias correction. *IEEE Trans Med Imaging*. 2010;29(6):1310-1320. doi:10.1109/TMI.2010.2046908
9. Coupe P, Yger P, Prima S, Hellier P, Kervrann C, Barillot C. An optimized blockwise nonlocal means denoising filter for 3-D magnetic resonance images. *IEEE Trans Med Imaging*. 2008;27(4):425-441. doi:10.1109/TMI.2007.906087
